# Prevalence of peripheral arterial disease in patients at non-high cardiovascular risk. Rationale and design of the PANDORA study

**DOI:** 10.1186/1471-2261-10-35

**Published:** 2010-08-05

**Authors:** Claudio Cimminiello, Claudio Borghi, Serge Kownator, Jean Claude Wautrecht, Christos P Carvounis, Stefanus E Kranendonk, Beat Kindler, Mario Mangrella

**Affiliations:** 1 Department of Medicine, via Cesare Battisti 23, Vimercate Hospital, Vimercate (MI), 20059, Italy; 2 Internal Medicine Unit, University Hospital Sant'Orsola-Malpighi, Bologna, Italy; 3 Clinique Ambroise Paré, Cardiology Department, Thionville, France; 4 Department of Vascular Diseases, Hôpital Erasme, Brussels, Belgium; 5 Internal Medicine Department, Blue Cross Hospital, Athens, Greece; 6 TweeSteden Hospital, Dr Deelenlaan 5, 5042 AD Tilburg, The Netherlands; 7 General Practitioner, Dufourstrasse 77, 8008 Zurich, Switzerland; 8 R&D Department, AstraZeneca SpA, Milan, Italy

## Abstract

**Background:**

Lower extremity peripheral arterial disease (PAD) is a marker of widespread atherosclerosis. Individuals with PAD, most of whom do not show typical PAD symptoms ('asymptomatic' patients), are at increased risk of cardiovascular ischaemic events. American College of Cardiology/American Heart Association guidelines recommend that individuals with asymptomatic lower extremity PAD should be identified by measurement of ankle-brachial index (ABI). However, despite its associated risk, PAD remains under-recognised by clinicians and the general population and office-based ABI detection is still poorly-known and under-used in clinical practice. The Prevalence of peripheral Arterial disease in patients with a non-high cardiovascular disease risk, with No overt vascular Diseases nOR diAbetes mellitus (PANDORA) study has a primary aim of assessing the prevalence of lower extremity PAD through ABI measurement, in patients at non-high cardiovascular risk, with no overt cardiovascular diseases (including symptomatic PAD), or diabetes mellitus. Secondary objectives include documenting the prevalence and treatment of cardiovascular risk factors and the characteristics of both patients and physicians as possible determinants for PAD under-diagnosis.

**Methods/Design:**

PANDORA is a non-interventional, cross-sectional, pan-European study. It includes approximately 1,000 primary care participating sites, across six European countries (Belgium, France, Greece, Italy, The Netherlands, Switzerland). Investigator and patient questionnaires will be used to collect both right and left ABI values at rest, presence of cardiovascular disease risk factors, current pharmacological treatment, and determinants for PAD under-diagnosis.

**Discussion:**

The PANDORA study will provide important data to estimate the prevalence of asymptomatic PAD in a population otherwise classified at low or intermediate risk on the basis of current risk scores in a primary care setting.

**Trial registration number:**

Clinical Trials.gov Identifier: NCT00689377.

## Background

Lower extremity peripheral arterial disease (PAD) is considered to be a marker of widespread atherosclerosis. In patients without overt cardiovascular disease (CVD), the presence of PAD predicts approximately a 30% risk of myocardial infarction, ischaemic stroke, and vascular death over 5 years. Patients with PAD show a 2- to 6-fold increase in death from cardiovascular causes and a greater risk of limb amputation than those without PAD. Individuals with PAD also experience higher 1-year rates of cardiovascular death (2.5%) and major cardiovascular events (21.1%) caused by an increase in atherothrombotic events, compared with patients with coronary artery disease (1.9% and 15.2%, respectively) or with any cerebrovascular disease (2.1% and 14.5%, respectively) [1-7].

Despite its association with severe health risk, PAD remains under-recognised by clinicians and the general population [8,9]. This may be due to the fact that the majority of individuals with lower extremity PAD do not experience recognisable ischaemic symptoms in the limb and are considered 'asymptomatic'. Nevertheless, these individuals share the unfavourable cardiovascular prognosis of symptomatic patients, as they show a risk profile comparable to that of patients with symptomatic lower extremity PAD or with coronary heart disease (CHD) [1,2,10,11]. Patients diagnosed with PAD, including those with asymptomatic PAD, should be treated to reach the recommended therapeutic goals, including low-density lipoprotein cholesterol (LDL-C) target levels <2.6 mmol/L (100 mg/dL) [12], which are similar to those recommended for high risk patients, i.e. those with CHD or CHD risk equivalents - although more challenging than that recommended for individuals at moderate CVD risk, i.e. 3.3 mmol/L (130 mg/dL) [2,12,13].

Ankle-brachial index (ABI) value ≤0.90 is the recognised cut-off for diagnosis of PAD. According to a few observational studies, pathological ABI values are frequently observed among elderly patients (aged 70+ years or 50-69 years but with a history of cigarette smoking) who are otherwise not classified as high risk [1,2]. Indeed, the prevalence of a pathological ABI still remains substantially unknown among patients classified at intermediate or low risk according to the current risk scores. The American College of Cardiology (ACC)/American Heart Association (AHA) guidelines recommend screening for both symptomatic and asymptomatic PAD, by physical examination and/or ABI measurement, in order to identify patients with asymptomatic lower limb PAD and to offer appropriate therapeutic interventions that diminish risk of myocardial infarction, stroke, and death.

Hence, the Prevalence of peripheral Arterial disease in patients with a non-high CVD risk, with No overt vascular Diseases nOR diAbetes mellitus (PANDORA) study was designed to provide updated information on the prevalence of PAD, in the context of traditional risk factor assessment, and insight on clinical characteristics as possible determinants for PAD under-diagnosis.

## Methods/Design

PANDORA is a non-interventional, cross-sectional, pan-European study of patients with a non-high CVD risk, defined as having at least two CVD risk factors according to National Cholesterol Education Program (NCEP) Expert Panel on Detection, Evaluation, and Treatment of High Blood Cholesterol in Adults (Adult Treatment Panel III [ATP III]) guidelines, with no overt CVDs or diabetes mellitus. Patients with definite symptoms of PAD will be also excluded (ClinicalTrials.gov Identifier: NCT00689377).

Approximately 1,000 sites will participate in the study in six European countries (Belgium, France, Greece, Italy, The Netherlands, Switzerland). Study objectives and selection criteria are listed in Table 1. Since the majority of individuals with a CVD risk factor score of 0 or 1 have a low 10-year risk, i.e. below 10%, these patients are excluded from the study population [13]. Study variables, as listed in Table 2, are collected in the Subject Record Form. Data are captured electronically in most participating countries.

**Table 1:** Study objectives and selection criteria

Primary objective	Secondary objectives
• To establish the prevalence of lower extremity PAD, defined as an ABI ≤0.9 in patients with at least two cardiovascular disease (CVD) risk factors, with no overt CVDs, including typical symptoms of PAD, or diabetes mellitus	• To establish the prevalence of PAD in the context of the cardiovascular risk level as assessed by the current CVD risk charts or algorithms
	• To establish the prevalence of CVD risk factors in patients with at least two CVD risk factors, with no overt CVDs or diabetes mellitus
	• To establish the risk factor treatment in patients with at least two CVD risk factors, with no overt CVDs or diabetes mellitus
	• To identify determinants (i.e. patient and physician characteristics) for PAD under-diagnosis, defined as the detection of an ABI ≤0.9 in a patient never diagnosed for PAD

**Inclusion criteria**	**Exclusion criteria**

• Either sex, any race	• Fewer than two risk factors for CVD
• Males aged ≥45 years or females aged ≥55 years (age-related CVD risk factor)	• Symptoms of PAD
• At least one additional risk factor for CVD, from the following:	• Type 1 or 2 diabetes mellitus
- cigarette smoking (any amount of tobacco smoked in the past month)	• CHD, including history of myocardial infarction, unstable angina, stable angina, coronary artery procedures (coronary artery bypass graft or percutaneous coronary intervention), or evidence of clinically significant myocardial ischaemia
- arterial blood hypertension (arterial blood pressure: ≥140 mmHg systolic and/or ≥90 mmHg diastolic or taking antihypertensive medication)	• CHD risk-equivalents, that include clinical manifestations of non-coronary forms of atherosclerotic disease, i.e. abdominal aortic aneurysm, or carotid artery disease (transient ischaemic attack or stroke)
- low high-density lipoprotein (HDL) cholesterol (<40 mg/dL, corresponding to <1.0 mmol/L) or high LDL cholesterol (≥130 mg/dL, corresponding to ≥3.3 mmol/L), within 3 months of study entry	• No blood lipid data collected in the last 12 months
- family history of premature coronary heart disease (CHD) before 55 years of age in father or other male first-degree relative, or before 65 years in mother or other female first-degree relative	• Serious or unstable medical or psychological conditions that, in the opinion of the Investigator, would compromise the patient's safety or successful participation in the study
- elevated waist circumference (≥102 cm for male; ≥88 cm for female)	• Patient who is unwilling or unable to provide informed consent
• Willingness to participate in the study and complying with the study by signing a written informed consent	

**Table 2:** Data captured in the Patient Record Form (PRF)

Date of birth, sex, race, civil status
Physical examination, i.e. height, weight, waist circumference, heart pulse, sitting blood pressure

Presence of CVD risk factors
• Cigarette/tobacco smoking habits
• Physical activity (a physically active patient is defined as an individual performing at least 30 minutes of continuous or intermittent moderate-intensity exercise 5 days/week)
• Alcohol intake
• Family history of premature CHD, as defined in Table 1
• Arterial blood hypertension, as defined in Table 1
• Dyslipidaemia (type of dyslipidaemia; whether untreated or on current lipid-lowering drug treatment)

Lipid data from fasting blood sample taken in the past 12 months (last measure) (date of the blood sample; total cholesterol, HDL cholesterol, and triglycerides as minimum requirements; LDL cholesterol, glucose, Apo-A1, and Apo-B, if available)

Current pharmacological treatments (drug Anatomical Therapeutic Chemical (ATC) class, duration of treatment, reason for therapy)

Right and left ABI measurement at rest, performed according to American College of Cardiology (ACC)/American Heart Association (AHA) guidelines for the management of patients with PAD

Prior to the inclusion of the first patient, investigators are requested to complete an Investigator Questionnaire regarding their personal experience and perception of the management of CVD risk, and attitude towards CVD diagnosis, guidelines and goals. The study will collect data on knowledge and behaviour in CVD diagnosis and management, quantity of CVD cases experienced, and attitudinal statements about guidelines and goals.

Patients recruited will be asked to complete a Patient Questionnaire which aims to collect data on awareness of CVD risk factors, knowledge and awareness of PAD, CVD risk perception and treatment, frequency of medical appointments.

The study conforms to the ethical principles outlined in the Declaration of Helsinki and is consistent with ICH/Good Clinical Practice, applicable regulatory requirements, and the AstraZeneca policy on Bioethics.

### ABI measurement

Each investigator receives theoretical and practical training on the ABI measurement technique according to ACC/AHA guidelines for the management of patients with PAD [1,2]. Right and left ABI measurement is performed at rest by measuring the systolic blood pressure from both brachial arteries and from both the dorsalis pedis and posterior tibial arteries after the patient has been at rest in the supine position for 10 minutes. Measurements are taken with blood pressure cuffs that are appropriately sized to the patient's lower calf (immediately above the ankle). The hand-held Doppler ultrasonography instrument Elite 100R, Nicolet Vascular Inc. (Golden, Colorado, USA), with an 8 MHz vascular probe, is used to measure systolic pressures and ABI. The Elite 100R vascular Doppler device was chosen since it has been used in major published studies on PAD detection [1,14]. The 8 MHz probe is preferred over the 5 MHz probe, due to its higher sensitivity and reliability in detecting both superficial and deep arteries.

For ABI calculations, the higher of the two brachial artery systolic pressures, and the higher of the dorsalis pedis and posterior tibial pressures in each ankle, are selected. The right and left ABI values are separately calculated by dividing the higher of the two ankle systolic pressures in that leg by the higher brachial artery systolic pressure. Calculated ABI values are recorded to two decimal places. Each patient is considered to have PAD if ABI in either leg is 0.90 or less [1,2,15].

### Statistical analysis

The statistical analyses will be descriptive in nature. Data will be presented as mean ± standard deviation or median with minimum and maximum. Summary data will be presented from all available data. Categorical data will be described by the number and percentage of patients in each category. Missing observations will be presented in tables as a separate category. The calculation of proportions will not include the missing category.

The primary objective of this survey is to determine the proportion of patients with pathological ABI, according to ACC/AHA guidelines for the management of patients with PAD. The overall sample size calculations were based on a confidence interval approach to ensure that the estimates will have adequate precision. A reasonable requirement on the precision is that the incidence should be determined within ± 1%, i.e. the length of the two-sided 99% confidence should not exceed 1 percentage value in each direction from the estimated value. Based on these calculations and expecting a 15% prevalence of individuals with pathological ABI in the selected population [14], the minimum sample size considered sufficient to meet the objectives of this protocol is 8,454 patients. However, to account for potential drop-outs, the survey plans to enrol 9,000 patients. Such sample size also ensures that the proportion reporting on the primary endpoint can be estimated with sufficient precision, on a 'by-country' basis, to represent the country-based heterogeneity of this population.

## Results and discussion

PAD, especially in the asymptomatic stage is common, and its prompt screening and treatment can reduce cardiovascular risk [2].

Although PAD shares the same risk factors of other CVDs and the guidelines recommend the same preventive treatments and therapeutic targets, there is low awareness of PAD (26% for PAD, compared with 74% for stroke, and 67% for coronary artery disease and heart failure, respectively) [8]. There appears to be greater awareness even of a rare disease such as Lou Gehrig's disease (36%), or conditions such as multiple sclerosis (42%) and cystic fibrosis (29%) [12].

The detection of asymptomatic people with an ABI ≤0.90 allows for the identification of a subgroup at higher cardiovascular risk than can be determined based on the usual CVD risk assessment. Diagnosis of asymptomatic PAD in such a population assigns these individuals to the 'secondary prevention risk level' [12].

ABI measurement is now accepted as a simple and reliable vascular test to assess leg perfusion. ABI testing is appropriate in populations targeted from the epidemiological database to be at risk for PAD. Among patients with no history of CVD or diabetes, ABI measurement is recommended in certain categories appearing to be at moderate CVD risk, including individuals at higher-risk ages, and with risk factors typically associated with PAD, such as cigarette smoking [2]. Quite recently, Cacoub *et al*. assessed the prevalence of PAD in a population of patients with two or more cardiovascular risk factors [16]. Diehm *et al*. provided data on the prevalence of PAD in a large population of unselected individuals aged ≥65 years from the primary care setting [17]. However, data on the prevalence of asymptomatic PAD in a large population otherwise classified at low or intermediate risk are lacking.

Office-based detection of ABI is the most cost-effective tool for lower extremity PAD detection and provides objective data both for the diagnosis in epidemiological surveys and in-office practice, and monitoring of efficacy of therapeutic interventions [2,18,19].

ACC/AHA guidelines state that the initial responsibility for the detection of lower extremity PAD should be with primary-care providers, because such providers are best positioned to determine an at-risk population and to initiate educational, lifestyle, and cardiovascular risk reduction therapies [2].

## Conclusions

Low awareness of PAD and scarce familiarity with the ABI technique seems to greatly undermine the diagnosis of asymptomatic PAD patients, who are at comparable risk to patients with CHD and should be appropriately treated. Unfortunately, most asymptomatic patients do not receive a diagnosis of PAD prior to the occurrence of a morbid or fatal ischaemic disease or event [1,2]. This is particularly true for individuals with asymptomatic PAD otherwise classified at low or intermediate risk on the basis of current risk scores. Information on the prevalence of asymptomatic PAD in such a setting is lacking. Improving PAD awareness among clinicians and the public is crucial for effective CVD prevention, early detection and integrated treatment. The PANDORA study will provide important pan-European data to estimate the prevalence of asymptomatic PAD in people with a non-high CVD risk in a primary care setting, by using the ABI technique, a risk-free gold-standard diagnostic test for PAD. This study may provide additional insights on identifying an at-risk segment of population that may benefit from appropriate preventive therapy.

## Abbreviations

ABI: ankle-brachial index; ATC: anatomical therapeutic chemical; CHD: coronary heart disease; CVD: cardiovascular disease; HDL: high-density lipoprotein; LDL: low-density lipoprotein; PAD: peripheral arterial disease; PANDORA: Prevalence of peripheral Arterial disease in patients with a non-high CVD risk, with No overt vascular Diseases nOR diAbetes mellitus; PCI: percutaneous coronary intervention.

## Competing interests

CC has no financial or non-financial competing interests. He has acted as a speaker and chairman at scientific meetings sponsored by AstraZeneca, Sanofi-Aventis and Bristol-Myers Squibb.

CB has acted as a consultant or speaker on occasions for Recordati, AstraZeneca, Merck, MSD, Novartis, Boehringer Ingelheim, Takeda, and Schering-Plough, and has received research funding from Boehringer Ingelheim, Sanofi-Aventis, Takeda, Italian Society of Hypertension, and the Regional Drug Agency. He holds shares in Abbott and Bristol-Myers Squibb and has received honoraria as a speaker in international and national meetings. He does not have any conflict of interest relating to the present activity.

SK has been a consultant or speaker for AstraZeneca, Boehringer-Ingelheim, Bristol-Myers Squibb, Daichi-Sankyo, MSD, Novartis, Pfizer, Sanofi-Aventis, Schering-Plough, and Solvay.

JCW has no financial or non-financial competing interests.

CPC has received honoraria from Mount Sinai Medical Center and has received research grants as National Coordinator of five Phase IV local studies. He has received grants and acted as a consultant for AstraZeneca.

SEK has acted as occasional speaker and has received honoraria as National Coordinator of the PANDORA study, and as consultant on PAD Advisory Board

BK has participated as investigator in an AstraZeneca sponsored clinical trial and has received honoraria

MM is an employee of AstraZeneca.

## Authors' contributions

CC participated in the study design, evaluated the statistical analysis, and drafted the manuscript. CB participated in the study design, evaluated the statistical analysis, and drafted the manuscript. SK participated in the study design and drafted the manuscript. JCW participated in the study design and drafted the manuscript. CPC participated in the study design and drafted the manuscript. SEK participated in the study design and drafted the manuscript. BK drafted, read and approved the final manuscript. MM participated in the study design, evaluated the statistical analysis, and drafted the manuscript. All authors read and approved the final manuscript.

## Pre-publication history

The pre-publication history for this paper can be accessed here:

http://www.biomedcentral.com/1471-2261/10/35/prepub
